# Trunk Muscle Activation Patterns During Standing Turns in Patients With Stroke: An Electromyographic Analysis

**DOI:** 10.3389/fneur.2021.769975

**Published:** 2021-11-11

**Authors:** I-Hsuan Chen, Pei-Jung Liang, Valeria Jia-Yi Chiu, Shu-Chun Lee

**Affiliations:** ^1^Department of Physical Therapy, Fooyin University, Kaohsiung City, Taiwan; ^2^Department of Rehabilitation Medicine, Taipei Tzu Chi Hospital, Buddhist Tzu Chi Medical Foundation, New Taipei City, Taiwan; ^3^School of Gerontology Health Management, College of Nursing, Taipei Medical University, Taipei City, Taiwan

**Keywords:** stroke, turning, electromyography, trunk muscles, muscle activation patterns

## Abstract

Recent evidence indicates that turning difficulty may correlate with trunk control; however, surface electromyography has not been used to explore trunk muscle activity during turning after stroke. This study investigated trunk muscle activation patterns during standing turns in healthy controls (HCs) and patients with stroke with turning difficulty (TD) and no TD (NTD). The participants with stroke were divided into two groups according to the 180° turning duration and number of steps to determine the presence of TD. The activation patterns of the bilateral external oblique and erector spinae muscles of all the participants were recorded during 90° standing turns. A total of 14 HCs, 14 patients with TD, and 14 patients with NTD were recruited. The duration and number of steps in the turning of the TD group were greater than those of the HCs, independent of the turning direction. However, the NTD group had a significantly longer turning duration than did the HC group only toward the paretic side. Their performance was similar when turning toward the non-paretic side; this result is consistent with electromyographic findings. Both TD and NTD groups demonstrated increased amplitudes of trunk muscles compared with the HC groups. Their trunk muscles failed to maintain consistent amplitudes during the entire movement of standing turns in the direction that they required more time or steps to turn toward (i.e., turning in either direction for the TD group and turning toward the paretic side for the NTD group). Patients with stroke had augmented activation of trunk muscles during turning. When patients with TD turned toward either direction and when patients with NTD turned toward the paretic side, the flexible adaptations and selective actions of trunk muscles observed in the HCs were absent. Such distinct activation patterns during turning may contribute to poor turning performance and elevate the risk of falling. Our findings provide insights into the contribution and importance of trunk muscles during turning and the association with TD after stroke. These findings may help guide the development of more effective rehabilitation therapies that target specific muscles for those with TD.

## Introduction

More than 40% of walking involves turning in daily life (1); however, turning frequently results in falls for patients with stroke (2). Patients with stroke require more time and additional steps (3) to turn in place (4, 5) or while walking (3, 6) compared with healthy adults, indicating that these patients experience difficulty in turning after stroke. Recent evidence indicates that the turning difficulty (TD) may be correlated with trunk control capacity (7, 8).

Lamontagne and colleagues employed motion analysis and observed that eye, head, and trunk rotations during walking turns in patients with subacute stroke were en bloc, and the patients did not exhibit intersegmental coordination (9). The simultaneous rotation of body segments may indicate axial or trunk instability during turning. By employing the Functional Assessment for Control of Trunk, Kobayashi and colleagues found that the time and number of steps required during 360° turning in place were strongly associated with trunk control (7). Similarly, Liang and colleagues reported that the duration of 180° walking turns was significantly associated with trunk control, as determined using the Trunk Impairment Scale (TIS) (8). In addition, our previous study indicated that turning toward the paretic side was associated with trunk flexion and rotation, trunk flexor strength, dynamic sitting balance, and trunk movement coordination (10). These findings highlight the importance of trunk function in turning performance and suggest that trunk instability contributes to the TD in individuals with stroke.

Compared with healthy adults, patients with stroke exhibited trunk muscle weakness including in the flexors, extensors, rotators, and lateral flexors of the trunk (11). Magnetic resonance imaging (12) and computer tomography (13) studies have revealed that the cross-sectional areas of trunk muscles were smaller after stroke than in healthy adults, indicating trunk paralysis. Weakness and spasticity are main motor impairments after stroke. Muscle weakness is primarily a result of damage to motor cortices and their descending corticospinal tract while medial reticulospinal tract hyperexcitability appears to be the most likely mechanism related to spasticity (14). In addition to trunk paralysis, trunk muscle spasticity, as well as limited trunk flexibility and abnormal position sense can further affect trunk function and motor control, as indicated by the dynamic balance subtest and the trunk rotation movement measure of the TIS (15).

The trunk contains the proximal and axial parts of the body, and its main function is to keep the body upright and maintain stability when performing static or dynamic activities. However, electromyography findings revealed that the trunk muscles of patients with stroke had slower onset latency and lower muscle amplitudes while standing and raising their arms (16). In addition, lower symmetric indexes in the internal oblique and rectus abdominal muscles during trunk flexion and in the erector spine muscles during trunk extension were observed (17), indicating trunk impairment after stroke. Furthermore, deficits in trunk muscles were significantly associated with balance problems, gait dysfunction, mobility impairment, dependency in the activities of daily living, and increased risk of falls (18). Although poor electromyographic performance of the trunk may be correlated with TD in patients with stroke, this correlation has not yet been investigated. Therefore, the current study evaluated the trunk muscle activation pattern during standing turns in healthy adults and patients with stroke with and without TD.

## Materials and Methods

### Participants

This prospective, cross-sectional study recruited patients with chronic stroke from the outpatient clinic of the department of physical and rehabilitation medicine of a regional hospital in New Taipei City, Taiwan, from June to November 2020. The study followed Strengthening the Reporting of Observational Studies in Epidemiology guidelines. The inclusion criteria were as follows: (1) survivors of a single and unilateral stroke with hemiparesis experienced for at least 6 months prior to participation in the study, (2) ability to walk independently over a distance of 10 m without requiring walking aids or orthoses, and (3) ability to provide informed consent and follow instructions. The exclusion criteria were (1) having an additional musculoskeletal condition or comorbid disability that could affect the assessment or (2) experiencing cognitive problems that were defined as having a Mini-Mental State Examination (MMSE) score of <24 or aphasia that could prevent patients from following instructions. Patients who received medical treatment and underwent rehabilitation were considered to have stable stroke conditions throughout the course of the study. Healthy controls (HCs) were recruited from the local community as the control group and were excluded if they had any neurological or musculoskeletal condition that could affect normal balance or the assessment procedure. All eligible participants provided written informed consent prior to participation in the study. This study was approved by the Institutional Review Board of Taipei Tzu Chi Hospital, Buddhist Tzu Chi Medical Foundation (Reference No. 08-XD-051) and registered on clinical.trials.gov (NCT04668573).

### Data Collection

Demographic data were recorded, namely age, sex, height, weight, and body mass index. Information regarding the poststroke duration, paretic side, lesion type, history of falls in the previous year, assistance devices, and rehabilitation frequency of patients with stroke was obtained, and their physical characteristics such as general cognitive function, lower-limb motor function, and functional mobility were evaluated. Subsequently, the 180° walking-turn performance of the patients with stroke was assessed, which were used to divide them into two groups according to the suggestion of Thigpen et al. (19). Finally, the activation pattern of the trunk muscles of the participants was evaluated during 90° standing turns. Taking one step to complete a 90° turn was chosen because one step made the beginning and end of turning easier to define and the patients with stroke experienced difficulty in completing a turn with a greater angle in one step, such as a 180° turn. All assessments were individually completed within 1 h by a well-trained research assistant.

### Measurements

#### General Cognitive Function

General cognitive function was evaluated using the MMSE (20), which assesses orientation to time and place, word registration, attention, calculation, recent word recall, language, and visual construction. Cognitive impairment was defined as an MMSE score of ≤ 24 points.

#### Lower-Limb Motor Function

Lower-limb motor function was defined according to the seven Brunnstrom classification stages: (1) flaccidity, (2) appearance of spasticity, (3) increased spasticity, (4) decreased spasticity, (5) complex movement combinations, (6) spasticity disappearance, and (7) return of normal function (21).

#### Functional Mobility

Functional mobility was assessed as balance, mobility, and walking function.

##### Functional Reach Test

Balance was assessed using the functional reach test (22). The participants stood close to the wall with their feet and shoulder width apart and non-paretic arms raised to 90° flexion. They reached as far forward as possible while maintaining their balance. A longer distance (cm) represented better balance.

##### Timed Up and Go Test

Mobility was examined using the timed up and go test (TUG) (23). The participants were instructed to stand up from a chair, walk 3 m, turn around, walk back to the chair, and sit down. The time (s) required to complete the task was recorded. Longer time was representative of a lower level of mobility.

##### Ten-Meter Walk Test

Walking function was measured using the 10-m walk test (24). The participants were asked to walk straight along a 14-m walkway at their fastest walking pace. The initial and final 2 m of the walkway were used for acceleration and deceleration. Only the time spent in the middle 10 m was recorded, and walking velocity (m/s) was calculated by dividing the walking distance by walking time. A faster walking velocity indicated better walking function.

#### Turning Performance

The participants were instructed to perform the TUG for the 180° walking-turn task. They were asked to rise from a chair, walk straight for 3 m, exceed a line, turn around (180°), walk back to the chair, and return to a seated position at a self-selected speed. The participants performed the task in each direction only once before one practice trial. We noted the direction in which the participants chose to turn and asked them to repeat the procedure in the opposite direction.

Turning performance was measured using APDM Opal wireless sensors and Mobility Lab software (APDM, Portland, OR, USA). The Opal is a lightweight (22 g) inertial sensor with a battery life of 16 h and 8 GB of storage. Three Opal inertial sensors were firmly attached to the participants by using elastic Velcro bands, with one on the middle lower back (5th lumbar vertebra process) and one on the top of each foot. Data were recorded at 128 Hz, stored in the internal memory of the Opal sensor, and uploaded later to a laptop computer. The duration (s) and number of steps (n) of the 180° turns were recorded. The horizontal rotational rate of the lumbar sensor was employed with a minimum of 45° accompanied with at least one right and one left foot stepping to detect turns (25, 26). The patients with stroke who required more than 3 s or five steps to complete a 180° walking turn were seen as poor turning performance and were included into the TD stroke group (19), whereas the remaining patients were seen as better turning performance and were included into the no TD (NTD) stroke group.

#### Muscle Activation Patterns

**Figure 1:**
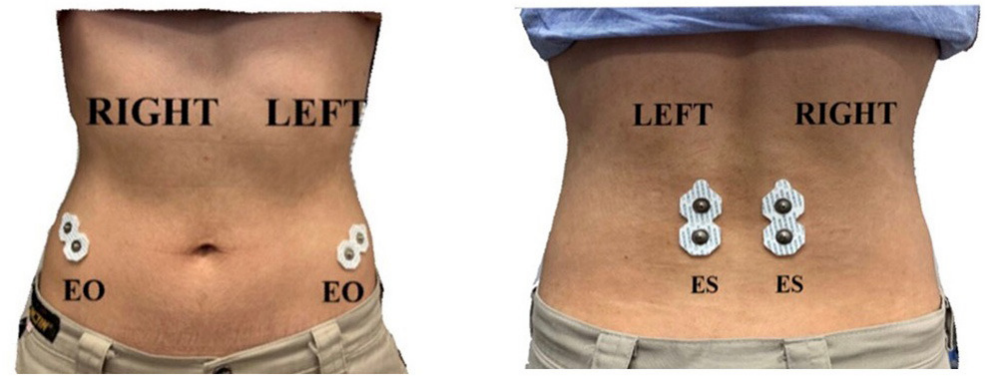
Placement of surface EMG electrodes. EO, external oblique muscle; ES, erector spinae.

Surface electromyography (EMG) data were obtained using a TeleMyo Mini DTS System (Noraxon USA, Inc., USA), with a sampling rate of 1,500 Hz. The skin was shaved and cleaned with alcohol swabs before applying disposable and self-adhesive Ag/AgCl snap surface electrodes (Noraxon USA, Inc., USA) for recording EMG data. The electrodes were positioned with an interelectrode distance of 2 cm. Due to technological restrictions, surface EMG signals were collected bilaterally (right and left) from the following trunk muscles and locations: external oblique (EO), ~15 cm lateral to the umbilicus, and erector spinae (ES), ~1 cm lateral to the L5 spinous process (27) (Figure 1). These muscles were chosen because they participate in the movement of trunk rotation (28). Because generating the maximal voluntary isometric contraction values in prone, supine, or side lying positions is difficult for patients with stroke (29, 30), normalization was performed using percentage reference voluntary contraction (RVCs). The RVCs of the trunk muscles for all the participants were tested in a sitting position on a bench with their legs bent and feet strapped down with a belt (31). One of the researchers provided a matching resistance to the participants during the maximal exertions to restrain their movement. To measure the RVC in the EO, the participants attempted to flex the upper trunk in the sagittal plane while their sternal notch was manually braced by the researcher. To measure the RVC in the ES, the participants attempted to extend the upper trunk in the sagittal plane, whereas their first thoracic vertebra spinous process was manually braced by the researcher. Each task was performed three times, and the resistance was applied for 6 s.

The participants were asked to stand with feet and shoulder width apart and arms at their sides and then to take one step to complete a standing 90° turn (standing and then turning to face the target) toward the paretic and non-paretic sides at a self-selected pace, corresponding to non-dominant and dominant sides, respectively, in HCs, in a counterbalanced order among participants. The dominant side of the HCs was determined by the side of the writing hand. Prior to testing, the researcher demonstrated the procedure to the participants. All the participants performed a practice trial to familiarize themselves with the test before three actual trials. The participants wore their regular footwear during testing. The EMG data of the four trunk muscles were recorded during turning and then averaged. We placed two accelerometers in bilateral shoe heels, the position corresponding to the calcaneus tuberosities, to determine the beginning and end of turning. Signals from trunk EMG and the accelerometer were simultaneously input into the software package for processing through a PC interface receiver (MyoResearch XP master, version 1.07.01, Noraxon USA, Inc., USA). All EMG data were bandpass filtered (10–500 Hz), full-wave rectified, and smoothed every 50 ms by using the root mean square. Muscle activity was normalized by RVC and is presented as the percent RVC.

### Statistical Analysis

Statistical analyses were performed using the Statistical Package for Social Sciences, version 19.0 (SPSS, Chicago, IL, USA). The statistical significance level was set at *p* < 0.05. Due to non-normally distributed data examined by the Shapiro–Wilk test, non-parametric tests were applied in the study. Data are presented as the number (percentage) and median (interquartile range). The Mann–Whitney U test or the Kruskal–Wallis test for continuous variables and the chi-squared test for categorical variables were applied to compare demographic data among the groups. The duration and number of steps in all turning tasks among the groups (TD stroke, NTD stroke, and HC) were analyzed using the Kruskal–Wallis test with *post-hoc* Mann–Whitney U test for between-group comparisons and the Wilcoxon signed-rank test for turning direction comparisons within groups. The Kruskal–Wallis test with *post-hoc* Mann–Whitney U test and the Friedman test with *post-hoc* Wilcoxon signed-rank test were used to compare the amplitude of trunk muscles between the groups and within the groups, respectively. In addition, we pooled all EMG data into SigmaPlot software (version 10.0, Systat Software Inc, San Jose City, CA, USA), which allowed us to create schematics that provided a visual comparison of changes in trunk muscle amplitudes over time during standing turns.

## Results

A total of 42 participants (14 HCs, 14 TD stroke, and 14 NTD stroke patients) were recruited (Table 1). No difference was observed among the groups in the demographic characteristics except for sex (*X*^2^ = 7.000, *p* = 0.03). The number of male patients with stroke was significantly higher than that of male HCs. The TD and NTD groups had similar ratios of paretic (P) side and lesion type, poststroke duration, cognitive function, lower-limb motor function, functional mobility, history of falls, use of assistance devices, and rehabilitation frequency.

**Table 1:** Demographic and physical characteristics of participants with stroke and healthy controls.

	**TD stroke**	**NTD stroke**	**Healthy controls**	***p*** **value**
	**(***n*** = 14)**	**(***n*** = 14)**	**(***n*** = 14)**	
Sex (male, n, %)	10 (71%)	10 (71%)	4 (29%)	**0.030**
Age (years)	55 (50–68)	55 (53–67)	62 (60–65)	0.275
Height (cm)	170 (158–174)	167 (159–174)	160 (157–170)	0.205
Weight (kg)	73 (65–81)	52 (68–76)	60 (53–76)	0.202
Body mass index (kg/m^2^)	26.0 (22.8–29.4)	23.5 (19.8–25.3)	24.3 (20.9–26.4)	0.400
Paretic side (left, n, %)	8 (57%)	8 (57%)		1.000
Post-stroke duration (month)	34 (14–140)	54 (26–109)		0.401
Lesion type (infarction, n, %)	4 (29%)	7 (50%)		0.440
Mini-mental state examination score (/30)	28 (25–29)	27 (25–28)		0.401
Brunnstrom stage-Leg (/6)	4 (4–4)	4 (3–5)		0.734
Functional reach test (cm)	18.0 (9.0–20.5)	18.0 (16.0–24.8)		0.306
Timed up and go test (s)	25.6 (17.3–36.7)	24.4 (16.1–24.8)		0.635
10-meter walk test (m/s)	0.6 (0.3–0.7)	0.6 (0.4–0.8)		0.667
History of falls (n, %)	11 (79%)	7 (50%)		0.236
Use of assistant devices (n, %)	12 (86%)	8 (57%)		0.209
Rehabilitation (hours per week)	3.5 (2.0–6.0)	3.5 (2.0–6.0)		0.839

*Values are presented as the number (percentage) and median (interquartile range, IQR). TD , turning difficulty; NTD, no turning difficulty. Significant difference was set as p < 0.05 and highlighted in bold*.

### Turning Duration and Steps

The TD stroke group exhibited a significantly longer duration and more number of steps than did the NTD stroke and HC groups when turning toward either side. The NTD group had a significantly longer turning duration toward the P side than did the HC group; however, the performance of the NTD group was similar to that of the HC group when turning toward the other side (Table 2). In terms of the turning direction, the TD stroke and HC groups exhibited similar turning performance toward either side, whereas the NTD stroke group used significantly more steps turning toward the P side than toward the non-paretic (NP) side (*p* = 0.036).

**Table 2:** Duration (s) and number of steps (n) for stroke participants with and without turning difficulty and healthy controls during 90° standing turns.

	**TD stroke**	**NTD stroke**	**HC**	**X^**2**^**	***p*** **value**	***post-ho*** **c analysis**	***p*** **^1^**	***p*** **^2^**	***p*** **^3^**
Turning toward paretic side									
Duration (s)	3.2 (2.9–3.4)	2.4 (2.0–2.7)	2.0 (1.8–2.2)	19.008	**<0.001**	TD > NTD > HC	**<0.001**	**0.001**	**0.014**
Number of steps (n)	4.5 (4.0–5.4)	3.0 (2.9–4.0)	3.8 (3.0–4.5)	14.695	**0.001**	TD > NTD	0.116	**0.017**	0.114
Turning toward non-paretic side									
Duration (s)	3.0 (2.7–3.6)	2.0 (1.7–2.7)	1.9 (1.8–2.1)	7.315	**0.026**	TD > NTD = HC	**<0.001**	**0.002**	0.667
Number of steps (n)	5.0 (3.5–5.5)	2.9 (2.0–3.6)	3.5 (3.0–4.0)	12.820	**0.002**	TD> NTD = HC	**0.009**	**0.001**	0.164

*Values are presented as median and interquartile range (IQR). Significant difference was set as p < 0.05 and highlighted in bold*.

*HC, healthy controls; TD, turning difficulty; NTD, no turning difficulty*.

*p^1^ = p value for difference between TD stroke and HC groups*.

*p^2^ = p value for difference between TD and NTD stroke groups*.

*p^3^ = p value for difference between NTD stroke and HC groups*.

### Muscle Activation Patterns in the Process of Turning Mobility

**Figure 2:**
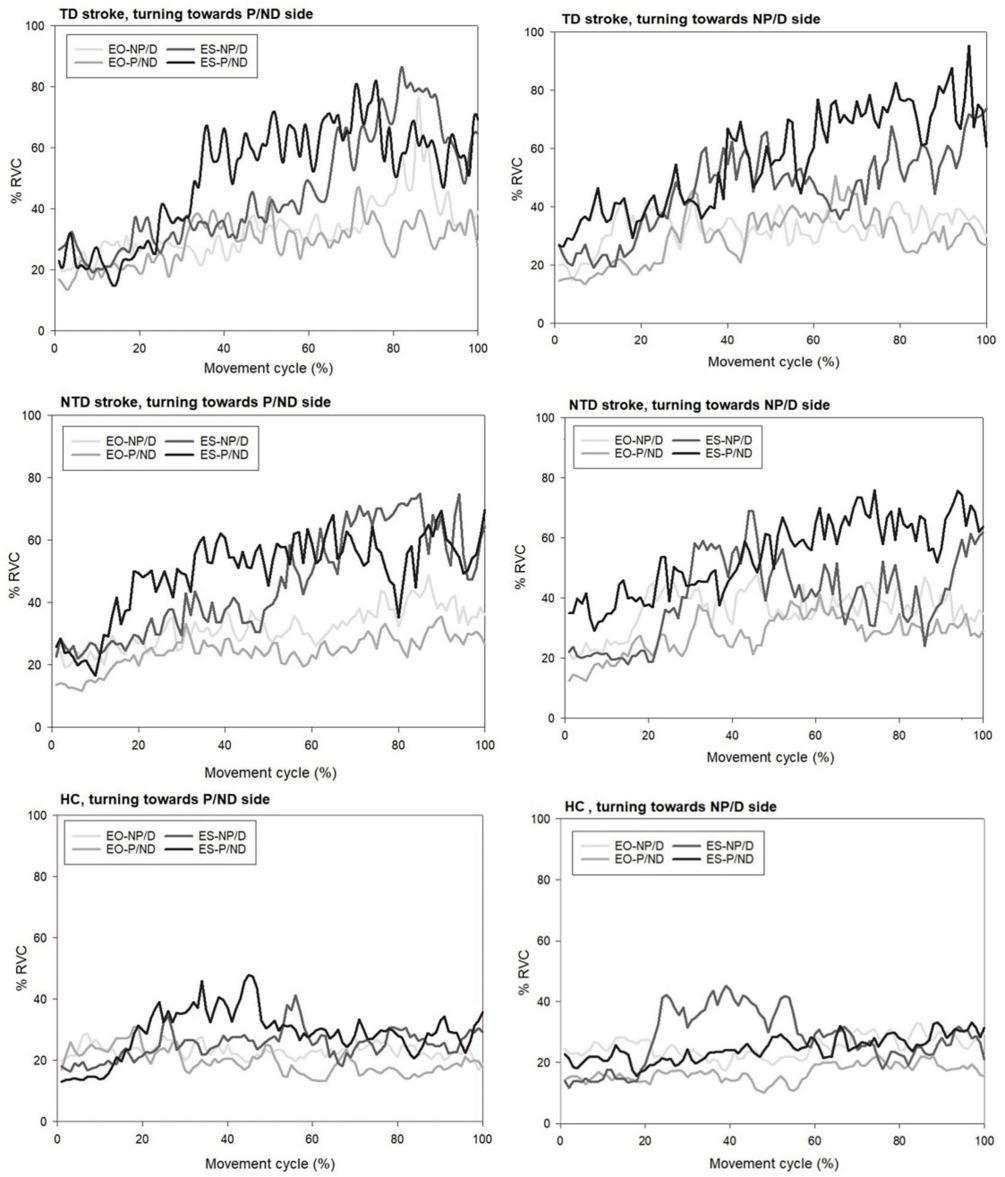
Representative plots of trunk muscle amplitudes for participants with stroke with and without turning difficulty and healthy controls when performing standing 90° turn tasks toward both sides. EO, external oblique muscle; ES, erector spinae; TD, turning difficulty; NTD, no turning difficulty; P, paretic side; NP, non-paretic side.

The muscle activation patterns of the groups during turning toward P and NP sides were compared visually by means of line graphs (Figure 2). The HC group exhibited stable and consistent contractions with an amplitude of approximately 20% RVC among the four trunk muscles throughout the entire movement of standing turns regardless of the turning direction. The amplitude in the ES of the turning side was increased in the beginning of turns but returned to the baseline after the midpoint. However, the TD group demonstrated gradually increased amplitudes in all the trunk muscles throughout the entire movement of standing turns, especially for bilateral ES muscles. The amplitude increased from 20 to 80% RVC of bilateral ES. A similar pattern was observed in the NTD group with the amplitude increasing from 20 to 60% RVC when turning toward the P side. The ES of the turning side was increased and then decreased afterward in the NTD stroke group when turning toward the NP side.

### Muscle Activation Patterns When Turning Toward the Paretic Side

The results of between-group analysis indicated significantly different muscle amplitudes in EO-P muscles [$X_{F}^{2}$ (2) = 6.731, *p* = 0.035] among the groups, with a higher amplitude in the TD group than in the HC group (*p* = 0.006). A significant difference was noted in ES-NP [$X_{F}^{2}$ (2) = 7.893, *p* = 0.019] muscle activity among the groups, with a higher amplitude in the TD (*p* = 0.004) and NTD (*p* = 0.041) groups than in the HC group. Significant differences in ES-P [$X_{F}^{2}$ (2) = 10.435, *p* = 0.005] muscle amplitudes were observed among the groups, with a higher amplitude in the TD group than in the HC group (*p* = 0.001). However, the amplitude of EO-NP among the groups was similar.

**Figure 3:**
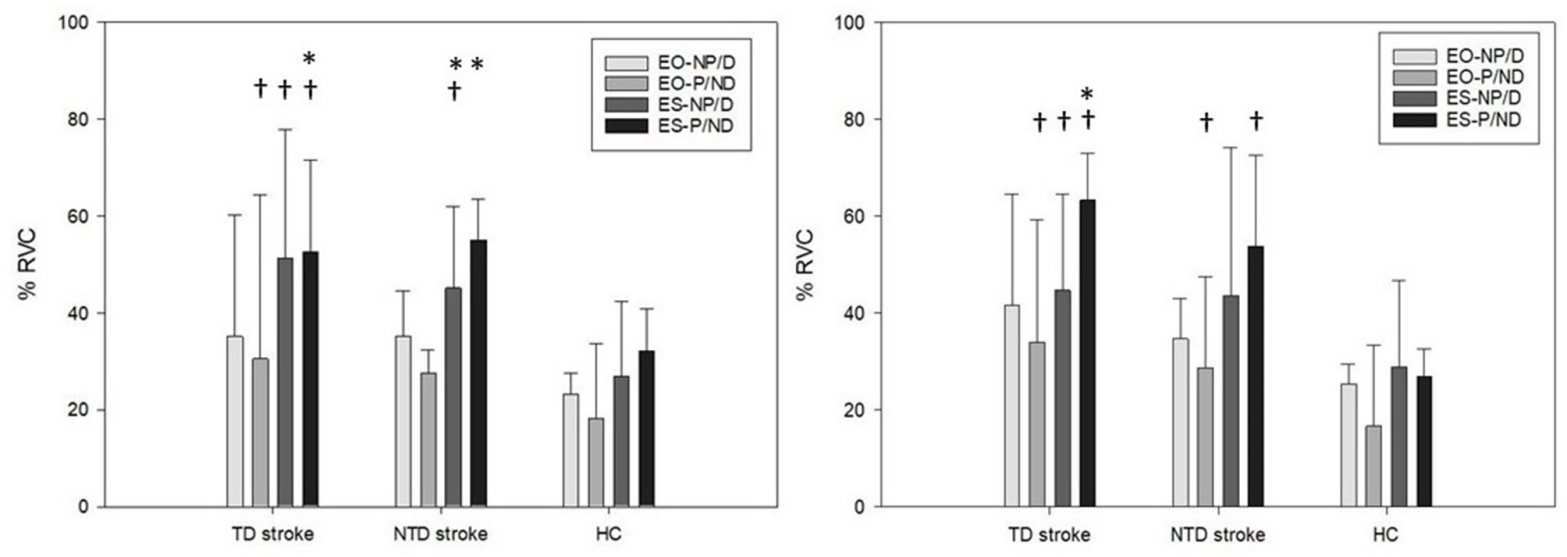
Bilateral external oblique and erector spinae muscles amplitudes (% RVC) for stroke participants with and without turning difficulties and healthy controls during 90° standing turns toward paretic (left) and non-paretic sides (right). EO, external oblique muscle; ES, erector spinae; TD, turning difficulty; NTD, no turning difficulty; P, paretic side; NP, non-paretic side; HC, healthy control. *represents a significant difference compared with EO. ^†^represents a significant difference compared with HCs.

The findings of within-group analysis indicated that both the TD [$X_{F}^{2}$ (3) = 11.571, *p* = 0.009] and NTD [$X_{F}^{2}$ (3) = 13.886, *p* = 0.003] groups had significantly different levels of muscle amplitudes among the trunk muscles, whereas the HC group had similar levels of amplitude among the trunk muscles. Further analysis demonstrated that the TD group had a higher amplitude in ES-P than in EO-NP (*p* = 0.005) and EO-P (*p* = 0.019) muscles, whereas the NTD group had a higher amplitude in ES-P and ES-NP compared with EO-NP (*p* = 0.022 and *p* = 0.005, respectively) and EO-P (*p* = 0.004 and *p* = 0.006) muscles (Figure 3).

### Muscle Activation Patterns When Turning Toward the Non-paretic Side

The results of between-group analysis revealed significantly different muscle amplitudes in EO-P muscles [$X_{F}^{2}$ (2) = 6.297, *p* = 0.043] among the groups, with a higher amplitude in the TD group (*p* = 0.041) and the NTD group (*p* = 0.023) than in the HC group. A significant difference in ES-NP [$X_{F}^{2}$ (2) = 6.073, *p* = 0.043] muscle activity was observed among the groups, with a higher amplitude in the TD than in the HC group (*p* = 0.013). Significant differences in ES-P [$X_{F}^{2}$ (2) = 12.201, *p* = 0.002] muscle amplitudes were observed among the groups, with a higher amplitude in the TD (*p* = 0.002) and NTD (*p* = 0.002) groups than in the HC group. However, the amplitudes for EO-NP among the groups were similar.

In within-group analysis, only the TD group [$X_{F}^{2}$ (3) = 10.200, *p* = 0.017] had significantly different levels of muscle amplitudes among the trunk muscles, with a higher amplitude in ES-P than in EO-P muscles (*p* = 0.016). The NTD and HC groups had similar levels of amplitude in trunk muscles (Figure 3).

## Discussion

This study investigated trunk muscle activation patterns during standing turns in healthy adults and patients with stroke with and without TD. The results indicated that the TD group exhibited a longer duration and required more steps to turn than did the HC group, independent of the turning direction. However, the NTD group exhibited a significantly longer turning duration toward the paretic side than did the HC group but their performance was similar when turning toward the non-paretic side. We observed that turning performance poststroke was related to trunk control capacity, as indicated by EMG findings. Both TD and NTD groups demonstrated an increased amplitude of trunk muscles compared with the HC group, and they failed to maintain consistent amplitudes among the four trunk muscles during the entire movement of standing turns in the direction that they required more time or steps to turn toward (i.e., turning in either direction for the TD group and turning to the paretic side for the NTD group). The results demonstrated the role of trunk control in standing turns, suggesting that trunk control capacity contributes to function recovery in turning after stroke.

In the healthy adults, the ES muscles of the turning side had a burst activity at 20–50% of the movement cycle, and then, a weak tonic activation of trunk muscles was maintained throughout the subsequent period during standing turns. The contralateral ES and bilateral EO muscles consistently demonstrated tonic contractions with amplitudes of ~20% RVC throughout the entire movement of standing turns regardless of the turning direction. Previous studies have reported that both the phasic and tonic periods of lumbar trunk muscle activity are required, and the activation of ES muscles is coordinated with the beginning stance of the contralateral leg (32, 33). The function of such anticipatory trunk muscle activity during functional tasks is to conserve trunk stability and to boost an inward tendency of body tilt toward the travel direction (32, 33). Limited studies have examined the role of trunk flexors in standing turns relative to trunk extensors; however, bilateral trunk flexors were observed to exhibit steady co-contraction with no burst activity preceding or during turning while pushing a cart (34). This finding suggests that bilateral trunk flexors steadily and cooperatively contract as anticipatory activation for the maintenance of core stability in response to turning by increasing trunk stiffness around the longitudinal axis (34).

We observed that patients with stroke demonstrated gradually increased amplitudes in all the trunk muscles throughout the entire movement of standing turns. The difference in the amplitude of the ES muscles between baseline and peak values even reached three to four times. Bilateral trunk flexors successfully co-contracted with weak tonic activation throughout the entire task, although the activation of paretic trunk flexors in the patients with stroke was greater than that in the HCs. After a stroke, the contractility of the trunk muscle is impaired and associated with balance ability and fall risk (35). We speculated that the augmented muscular activity establishes the core stability, which is a poststroke adaptation for balance maintenance and fall prevention because of higher challenges in dynamic balance during turns.

Such increased trunk muscle activity may compensate for the insufficient muscular recruitment of the lower extremities. The inner and outer legs normally play different roles during turns compared with linear walking. The inner leg must stabilize the posture, whereas the outer leg provides propulsion and swing to realign the body in the new direction (36). In our previous study, individuals with stroke had insufficient muscle activation in the tibialis anterior and biceps femoris of the paretic inner leg (36). Therefore, patients with stroke may increase the amplitude of trunk muscles to increase stiffness for greater trunk stability while turning toward the paretic side to compensate for poorer medial–lateral stability of the paretic leg working as the inner leg. Moreover, patients with stroke failed to selectively flex the hip and knee joints of the paretic outer leg and presented poor ground clearance while turning toward the non-paretic side (37). Patients with stroke compensate for the poor motor control of lower limbs by recruiting trunk muscles, such as a larger trunk rotation might be a compensation for limited hip flexion, or trunk leaning toward the paretic side for a pelvic drop at the swing leg (38). Although patients with stroke can perform standing turns, such malfunctioning movement patterns may result in the wastage of energy and inefficient performance (as was seen in the longer duration and greater numbers of steps relative to age-matched healthy adults). Future research should examine the kinematic characteristics of the trunk in standing turns after stroke.

Although muscles in the trunk and lower limb were impaired after stroke, their performance and EMG activity appeared to differ during turning tasks. The level of muscle activation was lower in the paretic lower limb (30) and higher in trunk muscles. A possible explanation is the distinct recovery process of neuromuscular pathways between muscles in the trunk and limbs. A recent transcranial magnetic stimulation study reported the role of the compensatory activation of uncrossed pathways from the non-paretic hemisphere in the recovery of trunk function (39). Other studies have suggested that the ipsilateral pathways of the non-paretic hemisphere would not be helpful in the motor recovery of the upper extremity (40). Trunk muscle performance is usually considered to be less affected after stroke than the performance of the upper and lower extremities and perhaps could contribute to the maintenance of whole-body balance under demanding equilibrium conditions during standing turns.

Our study goes beyond previous research with its comparison of patients with stroke and healthy adults and its analysis of turning performance and trunk muscle activation patterns in patients with stroke with and without TD. Most previous studies have reported a longer duration and greater numbers of step to complete a turn for patients with stroke (3–5, 41), and the performance was similar whether turning toward the paretic or non-paretic side (7, 42, 43). However, these results were observed only in the TD group in the current study; the NTD group turned differently according to the direction. They spent a longer duration than did the HC group when turning toward the paretic side, but their performance was similar to that of the HC group when turning toward the non-paretic side. This direction-specific difference was supported by our EMG findings. The NTD group demonstrated co-activation among the four trunk muscles while turning toward the non-paretic side, which was similar to the activation pattern observed in the HCs. However, more muscle activation of bilateral trunk extensors than trunk flexors was observed while turning toward the paretic side; this was similar to the activation pattern seen in the TD group. The compensation of trunk extensors may be used to provide a supportive function for the paretic leg to maintain balance. Such compensation could contribute to the quicker time and fewer steps to turn, but it may elevate the risk of falling. In a previous study, most falls among patients with stroke were reported to occur during turning to the paretic side (44). Our previous study also indicated that patients with stroke experienced greater difficulty turning toward the paretic side due to having a longer duration and reduced center of gravity displacement (10). We suggest that therapeutic rehabilitation programs include turning training for patients with stroke with and without TD, and turning toward both sides should be practiced. Investigating the direction-specific risk of falls during turning would also be useful with further subgroup analysis based on patients with stroke with or without TD.

A study reported a non-linear U-shaped relationship between the walking speed and fall rate (45). Such a relationship seems to also be present in turning tasks. Patients with stroke with slower turns with multiple steps may simply be walking more carefully to prevent falling. Patients with stroke with inadequate duration and numbers of steps in turning may not have sufficient balance control to successfully complete a turn. Therefore, in addition to turn duration and steps, muscular components during standing turns toward either direction should be considered in stroke rehabilitation to improve turning performance and prevent falls.

Previous studies have indicated that the motor control of the lower limb, balance ability, and cognitive function could contribute to turning performance after stroke (7, 42, 46). We did not observe significant differences in general cognitive function or functional mobility lower-limb motor function between the TD and NTD groups. Successful turning may require more motor recovery of trunk control than expected. Our findings highlight the role of trunk control in turning and neuromuscular strategies in stroke patients with and without TD.

This study has limitations. Only patients with chronic stroke who were able to walk a distance of 10 m without walking aids or orthoses were recruited. Caution should be taken when generalizing the results and conclusions. Moreover, due to technological restrictions, we examined only the activation patterns of four principal trunk muscles that identify the function of the lower back during standing turns. More trunk muscles should be included to comprehensively understand neuromuscular control in the trunk during turning. In addition, the increased trunk muscle activity observed in this study represents the greater muscle contraction, but could not exclude the possibility of hypertonic interference. Although our stroke patients had relatively decreased spasticity for paretic leg (median stage 4 in Brunnstrom classification) and may have similar observation in trunk, trunk muscle tone was not measured in the current study and future research can take this into consideration. The classification criteria based on suggestion of Thigpen et al. may not perfectly identify patients with TD from NTD because the development of the criteria was derived from the turning performance of the elderly. However, there are currently no other criteria for differentiating stroke patients with and without TD, and thus additional work is required to find more precise cut-off points. Finally, we chose a standing turn of 90° as the target task. To provide the whole picture regarding the role of trunk control in the directional change while walking, varied turning tasks including different turn angles or different circumstances could be considered in future studies.

## Conclusions

The main function of the trunk is to keep the body upright and maintain stability during static or dynamic activities. Turning is a challenging task for patients with stroke because of the requirement for side-dependent modulation of the legs and demanding trunk control to maintain balance. To the best of our knowledge, this is the first study to compare the activation patterns of the trunk flexors and extensors between healthy adults and patients with stroke with and without TD during standing turns. We observed augmented activation of trunk muscles in patients with stroke relative to HCs. When the TD group turned toward either direction and when the NTD group turned toward the paretic side, the flexible adaptations and selective actions of trunk muscles seen in HCs were absent. Such distinct activation patterns between patients and age-matched controls during standing turns could contribute to poor turning performance and may elevate the risk of falling in patients with stroke. The results provide insights into the contribution and importance of the trunk muscles during turning and the association with TD after stroke, which may help guide the development of more effective rehabilitation therapies that target specific muscles for those with TD.

## Data Availability Statement

The raw data supporting the conclusions of this article will be made available by the authors, without undue reservation.

## Ethics Statement

This study was approved by the Institutional Review Board of Taipei Tzu Chi Hospital, Buddhist Tzu Chi Medical Foundation (Reference No. 08-XD-051). The patients/participants provided their written informed consent to participate in this study.

## Author Contributions

SCL was a major contributor in study design. PJL and VJYC have done the data collection. IHC and SCL analyzed and interpreted data. IHC, PJL, and SCL have done the manuscript writing. All authors read and approved the final manuscript.

## Funding

This work was supported by the Taipei Tzu Chi Hospital and Buddhist Tzu Chi Medical Foundation (TCRD-TPE-109-09).

## Conflict of Interest

The authors declare that the research was conducted in the absence of any commercial or financial relationships that could be construed as a potential conflict of interest.

## Publisher's Note

All claims expressed in this article are solely those of the authors and do not necessarily represent those of their affiliated organizations, or those of the publisher, the editors and the reviewers. Any product that may be evaluated in this article, or claim that may be made by its manufacturer, is not guaranteed or endorsed by the publisher.

## References

[1] Glaister BC Bernatz GC Klute GK Orendurff MS. Video task analysis of turning during activities of daily living. Gait Posture. (2007) 25:289–94. 10.1016/j.gaitpost.2006.04.003 16730441

[2] Hyndman D Ashburn A Stack E. Fall events among people with stroke living in the community: circumstances of falls and characteristics of fallers. Arch Phys Med Rehabil. (2002) 83:165–70. 10.1053/apmr.2002.28030 11833018

[3] Leigh HK Hollands MA Zietz D Miles WA Wright C Van Vliet P. Kinematics of turning 180 degrees during the timed up and go in stroke survivors with and without falls history. Neurorehabil Neural Repair. (2010) 24:358–67. 10.1177/1545968309348508 19822720

[4] Ahmad RY Ashburn A Burnett M Samuel D Verheyden G. Sequence of onset latency of body segments when turning on-the-spot in people with stroke. Gait Posture. (2014) 39:841–6. 10.1016/j.gaitpost.2013.11.009 24326233

[5] Shiu CH Ng SS Kwong PW Liu TW Tam EW Fong SS. Timed 360 degrees turn test for assessing people with chronic stroke. Arch Phys Med Rehabil. (2016) 97:536–44. 10.1016/j.apmr.2015.11.010 26694578

[6] Danielli Coelho de. Morais Faria C, Fuscaldi Teixeira-Salmela L, Nadeau S. Effects of the direction of turning on the timed up & go test with stroke subjects. Top Stroke Rehabil. (2009) 16:196–206. 10.1310/tsr1603-196 19632964

[7] Kobayashi M Takahashi K Sato M Usuda S. Association of performance of standing turns with physical impairments and walking ability in patients with hemiparetic stroke. J Phys Ther Sci. (2015) 27:75–8. 10.1589/jpts.27.75 25642042 PMC4305603

[8] Liang PJ Chen JY Lee SC. The percentage of occurrence of turning difficulty in hemiplegic stroke survivors. J Physiother Res. (2018) 2:13.

[9] Lamontagne A Fung J. Gaze and postural reorientation in the control of locomotor steering after stroke. Neurorehabil Neural Repair. (2009) 23:256–66. 10.1177/1545968308324549 19060133

[10] Liang PJ Chiu VJ Teng YC Chiu HL Lee SC. Turning difficulties after stroke and its relationship with trunk function. Eur J Phys Rehabil Med. (2021). 10.23736/S1973-9087.21.06841-6. [Epub ahead of print]. 34042411

[11] Karthikbabu S Chakrapani M Ganeshan S Rakshith KC Nafeez S Prem V. Review on assessment and treatment of the trunk in stroke: a need or luxury. Neural Regen Res. (2012) 7:1974–7. 10.3969/j.issn.1673-5374.2012.25.008 25624827 PMC4298892

[12] Gibbons LE Latikka P Videman T Manninen H Battie MC. The association of trunk muscle cross-sectional area and magnetic resonance image parameters with isokinetic and psychophysical lifting strength and static back muscle endurance in men. J Spinal Disord. (1997) 10:398–403. 10.1097/00024720-199710000-00007 9355056

[13] Suzuki M Sonoda S Saitoh E Tsujiuchi K Hirose M Kamata Y. Paravertebral muscle area measurement in hemiplegics using computed tomography. Japanese J Rehabil Med. (1996) 33:176–81. 10.2490/jjrm1963.33.176

[14] Li S Francisco GE Zhou P. Post-stroke hemiplegic gait: new perspective and insights. Front Physiol. (2018) 9:1021. 10.3389/fphys.2018.01021 30127749 PMC6088193

[15] Lee Y An S Lee G. Clinical utility of the modified trunk impairment scale for stroke survivors. Disabil Rehabil. (2018) 40:1200–5. 10.1080/09638288.2017.1282990 28637127

[16] Dickstein R Shefi S Marcovitz E Villa Y. Anticipatory postural adjustment in selected trunk muscles in poststroke hemiparetic patients. Arch Phys Med Rehabil. (2004) 85:261–7. 10.1016/j.apmr.2003.05.011 14966711

[17] Liao CF Liaw LJ Wang RY Su FC Hsu AT. Electromyography of symmetrical trunk movements and trunk position sense in chronic stroke patients. J Phys Ther Sci. (2015) 27:2675–81. 10.1589/jpts.27.2675 26504267 PMC4616068

[18] Cabanas-Valdes R Cuchi GU Bagur-Calafat C. Trunk training exercises approaches for improving trunk performance and functional sitting balance in patients with stroke: a systematic review. NeuroRehabilitation. (2013) 33:575–92. 10.3233/NRE-130996 24018373

[19] Thigpen MT Light KE Creel GL Flynn SM. Turning difficulty characteristics of adults aged 65 years or older. Phys Ther. (2000) 80:1174–87. 10.1093/ptj/80.12.1174 11087304

[20] Folstein MF Folstein SE McHugh PR. ‘Mini-mental state’. A practical method for grading the cognitive state of patients for the clinician. J Psychiatr Res. (1975) 12:189–98. 10.1016/0022-3956(75)90026-6 1202204

[21] Brunnstrom S. Motor testing procedures in hemiplegia: based on sequential recovery stages. Phys Ther. (1966) 46:357–375. 10.1093/ptj/46.4.357 5907254

[22] Duncan PW Weiner DK Chandler J Studenski S. Functional reach: a new clinical measure of balance. J Gerontol. (1990) 45:M192–7. 10.1093/geronj/45.6.M192 2229941

[23] Podsiadlo D Richardson S. The timed ‘Up & Go’: a test of basic functional mobility for frail elderly persons. J Am Geriatr Soc. (1991) 39:142–8. 10.1111/j.1532-5415.1991.tb01616.x 1991946

[24] Flansbjer UB Holmback AM Downham D Patten C Lexell J. Reliability of gait performance tests in men and women with hemiparesis after stroke. J Rehabil Med. (2005) 37:75–82. 10.1080/16501970410017215 15788341

[25] Pearson S Mancini M El-Gohary M McNames J Horak FB. Turn detection and characterization with inertial sensors. In: icSPORTS. Vilamoura (2013). p. 19–22.

[26] El-Gohary M Pearson S McNames J Mancini M Horak F Mellone S. Continuous monitoring of turning in patients with movement disability. Sensors. (2014) 14:356–69. 10.3390/s140100356 24379043 PMC3926561

[27] Brown SHM Vera-Garcia FJ McGill SM. Effects of abdominal muscle coactivation on the externally preloaded trunk: variations in motor control and its effect on spine stability. Spine (Phila. Pa. 1976). (2006) 31:E387–93. 10.1097/01.brs.0000220221.57213.25 16741438

[28] Torén A. Muscle activity and range of motion during active trunk rotation in a sitting posture. Appl Ergon. (2001) 32:583–91. 10.1016/S0003-6870(01)00040-0 11703044

[29] Sousa ASP Tavares JMRS. Surface Electromyographic Amplitude Normalization Methods: A Review. Electromyography: New Developments, Procedures and Applications. (2012).

[30] Park BS Noh JW Kim MY Lee LK Yang SM Lee WD. Randomized controlled pilot trial of truncal exercises after stroke to improve gait and muscle activity. Neurosci Med. (2016) 7:149–56. 10.4236/nm.2016.74015

[31] Martins JC Teixeira-Salmela LF Lara EM Moura JB Aguiar LT de Faria CDCM. Validity and reliability of the modified sphygmomanometer test to assess strength of the lower limbs and trunk muscles after stroke. J Rehabil Med. (2014) 46:620–8. 10.2340/16501977-1823 24849895

[32] Courtine G Papaxanthis C Schieppati M. Coordinated modulation of locomotor muscle synergies constructs straight-ahead and curvilinear walking in humans. Exp Brain Res. (2006) 170:320–35. 10.1007/s00221-005-0215-7 16328271

[33] Hase K Stein RB. Turning strategies during human walking. J Neurophysiol. (1999) 81:2914–22. 10.1152/jn.1999.81.6.2914 10368408

[34] Lee YJ Hoozemans MJM. vanDieën JH. Trunk muscle control in response to (un) expected turns in cart pushing. Gait Posture. (2012) 36:133–8. 10.1016/j.gaitpost.2012.02.005 22406290

[35] Kim Y Kim J Nam H Kim HD Eom MJ Jung SH. Ultrasound imaging of the trunk muscles in acute stroke patients and relations with balance scales. Ann Rehabil Med. (2020) 44:273. 10.5535/arm.19125 32721990 PMC7463119

[36] Chen IH Yang YR Cheng SJ Chan RC Wang RY. Neuromuscular and biomechanical strategies of turning in ambulatory individuals post-stroke. Chin J Physiol. (2014) 57:128–36. 10.4077/CJP.2014.BAC204 24826781

[37] Rao N Aruin AS. Role of ankle foot orthoses in functional stability of individuals with stroke. Disabil Rehabil Assist Technol. (2016) 11:595–8. 10.3109/17483107.2015.1027300 25826046

[38] Van Criekinge T Saeys W Hallemans A Velghe S Viskens PJ Vereeck L. Trunk biomechanics during hemiplegic gait after stroke: a systematic review. Gait Posture. (2017) 54:133–43. 10.1016/j.gaitpost.2017.03.004 28288334

[39] Fujiwara T Sonoda S Okajima Y Chino N. The relationships between trunk function and the findings of transcranial magnetic stimulation among patients with stroke. J Rehabil Med. (2001) 33:249–55. 10.1080/165019701753236428 11766953

[40] Hoyer EH Celnik PA. Understanding and enhancing motor recovery after stroke using transcranial magnetic stimulation. Restor Neurol Neurosci. (2011) 29:395–409. 10.3233/RNN-2011-0611 22124033 PMC3613277

[41] Faria CDCM Reis DA Teixeira-Salmela LF Nadeau S. Performance of hemiplegic patients in 180° turns in the direction of the paretic and non-paretic sides before and after a training program. Rev Bras Fisioter. (2009) 13:451–9. 10.1590/S1413-35552009005000052

[42] Lam T Luttmann K. Turning capacity in ambulatory individuals poststroke. Am J Phys Med Rehabil. (2009) 88:873–83. 10.1097/PHM.0b013e3181bc0ddf 19893383

[43] Robinson RL Ng SSM. The timed 180 degrees turn test for assessing people with hemiplegia from chronic stroke. Biomed Res Int. (2018) 2018:9629230. 10.1155/2018/9629230 29568774 PMC5820648

[44] Cumming RG Klineberg RJ. Fall frequency and characteristics and the risk of hip fractures. J Am Geriatr Soc. (1994) 42:774–8. 10.1111/j.1532-5415.1994.tb06540.x 8014355

[45] Quach L Galica AM Jones RN Procter-Gray E Manor B Hannan MT. The nonlinear relationship between gait speed and falls: the maintenance of balance, independent living, intellect, and zest in the elderly of Boston study. J Am Geriatr Soc. (2011) 59:1069–73. 10.1111/j.1532-5415.2011.03408.x 21649615 PMC3141220

[46] Manaf H Justine M Goh HT. Axial segmental coordination during turning: effects of stroke and attentional loadings. Motor Control. (2017) 21:42–57. 10.1123/mc.2015-0040 26595318

